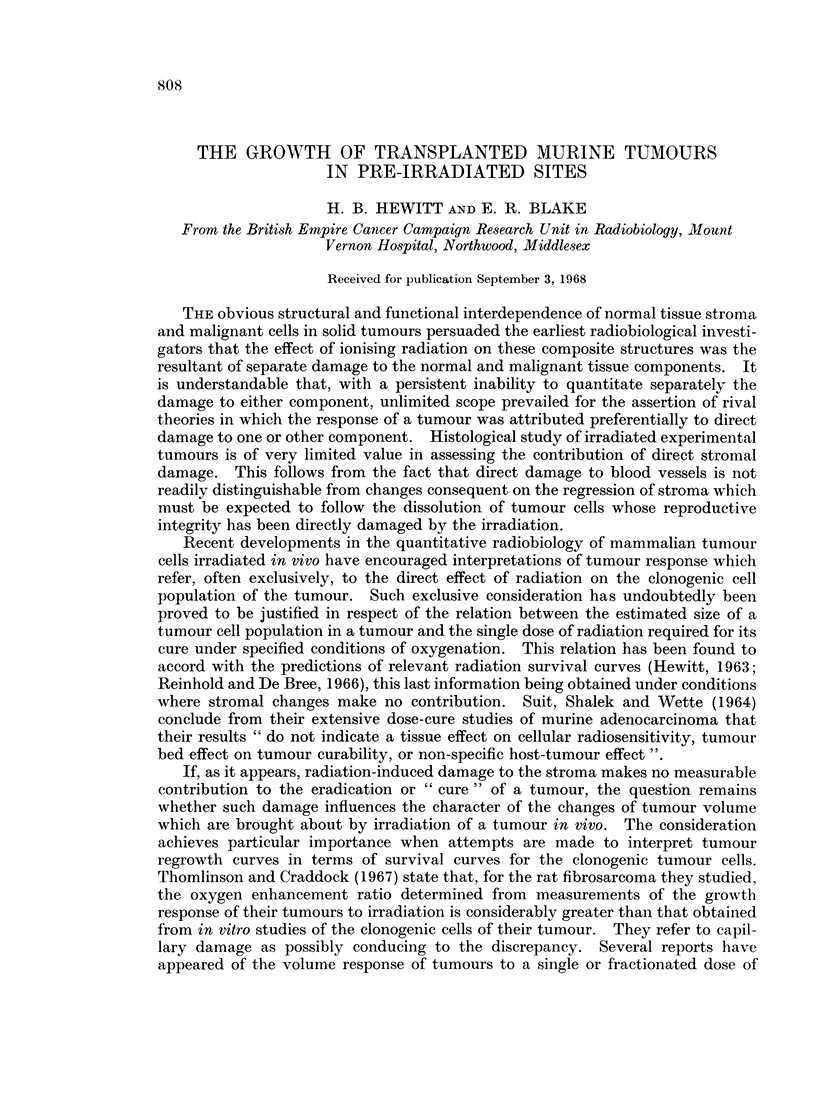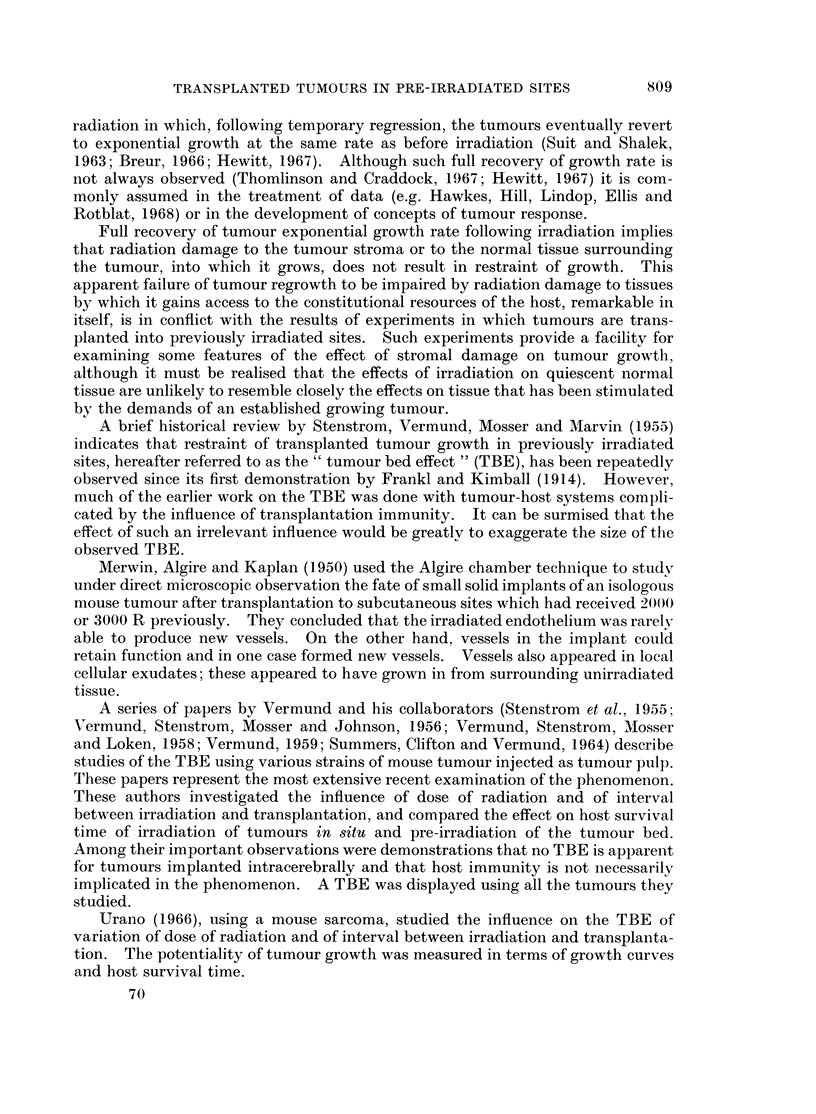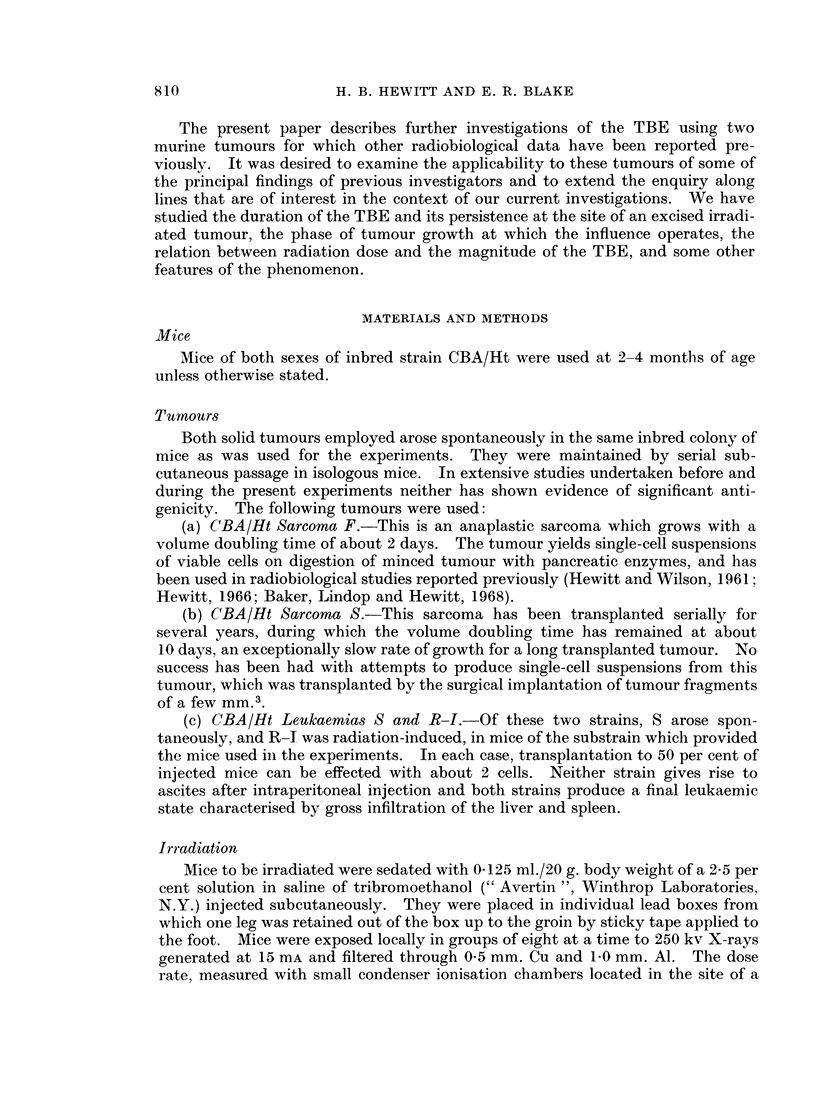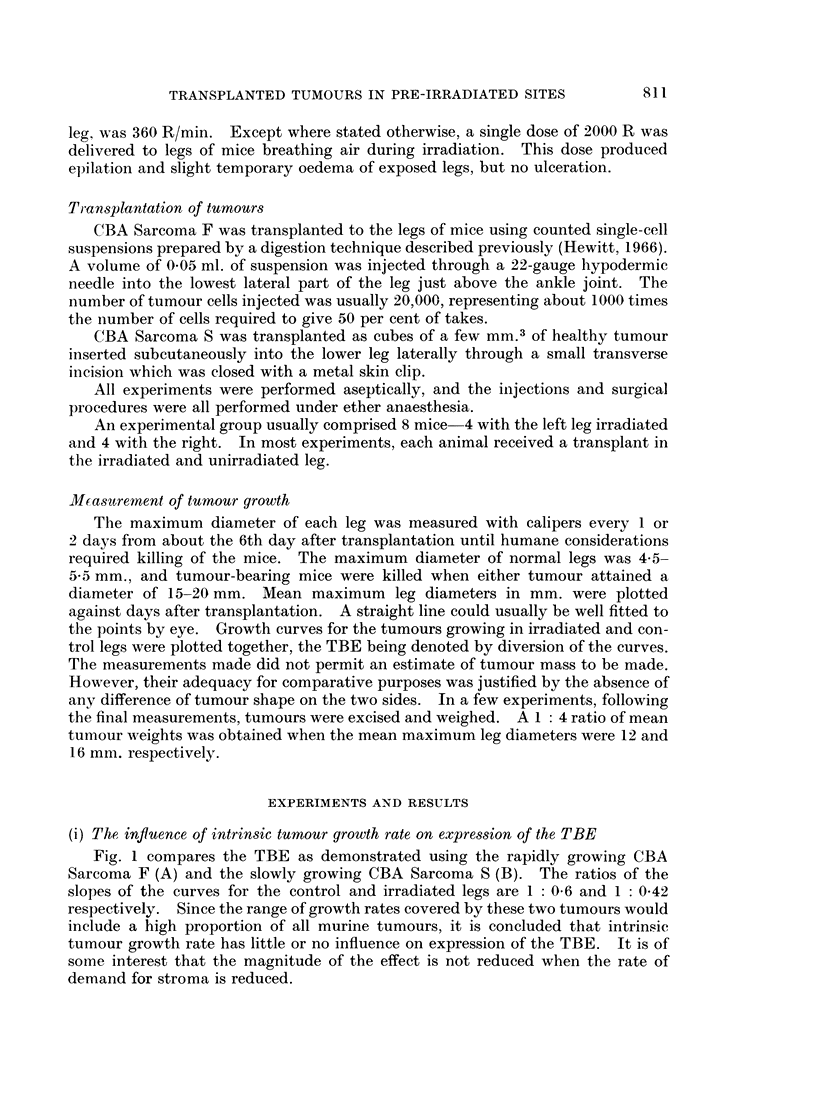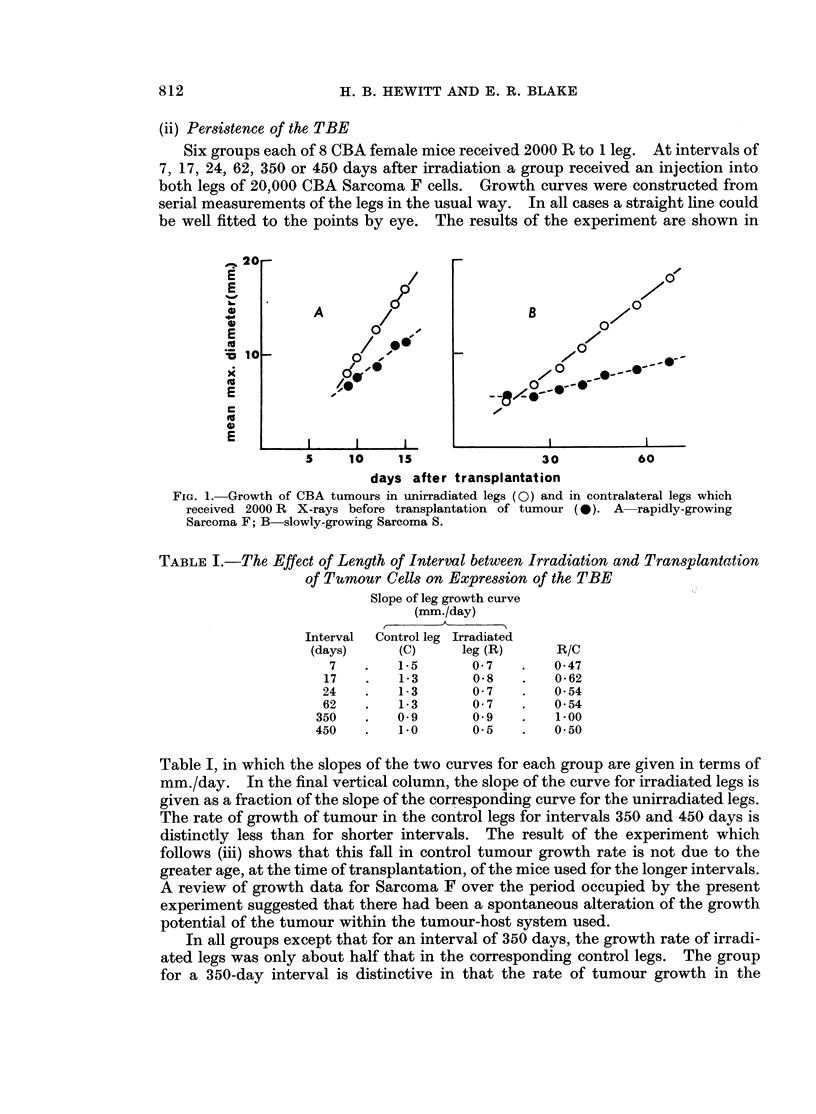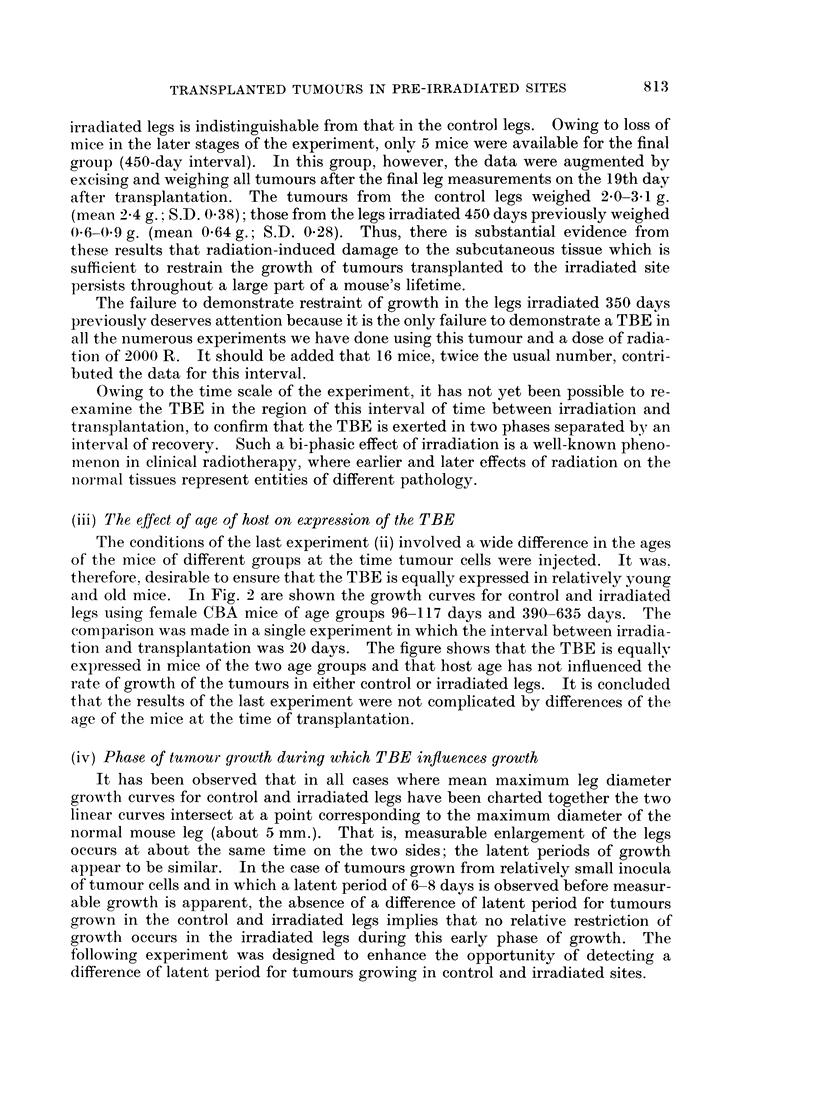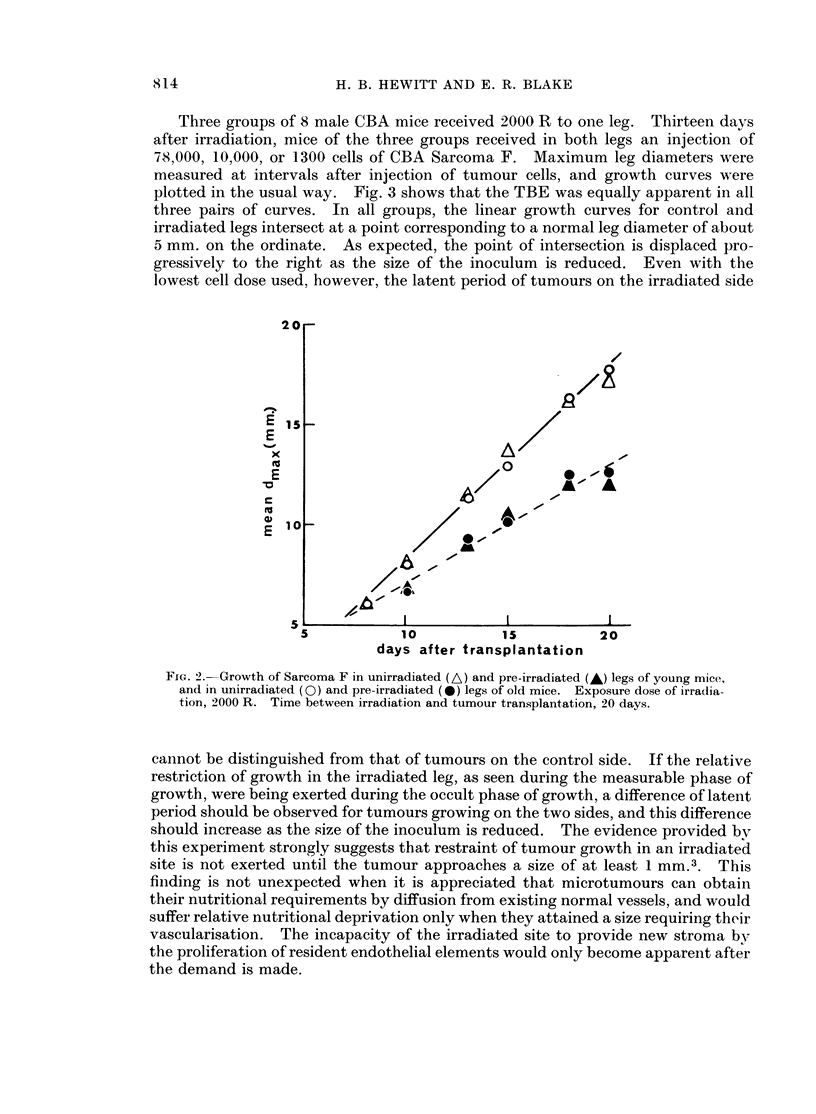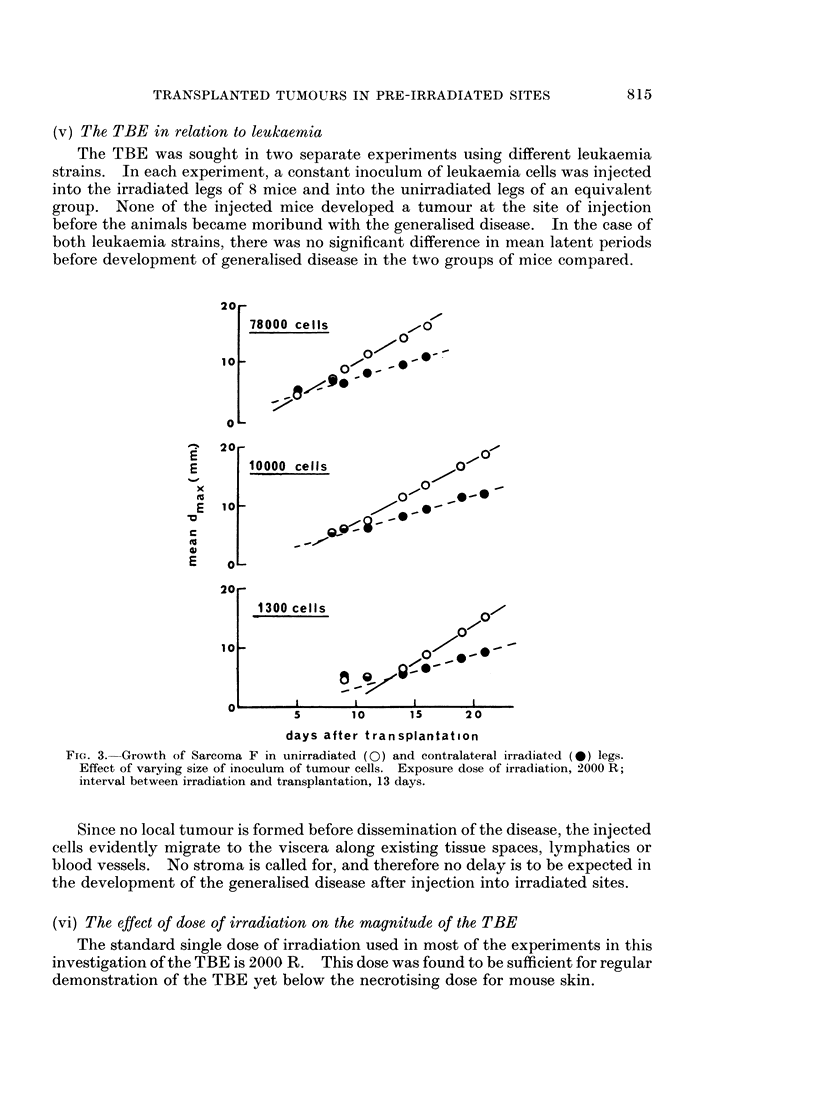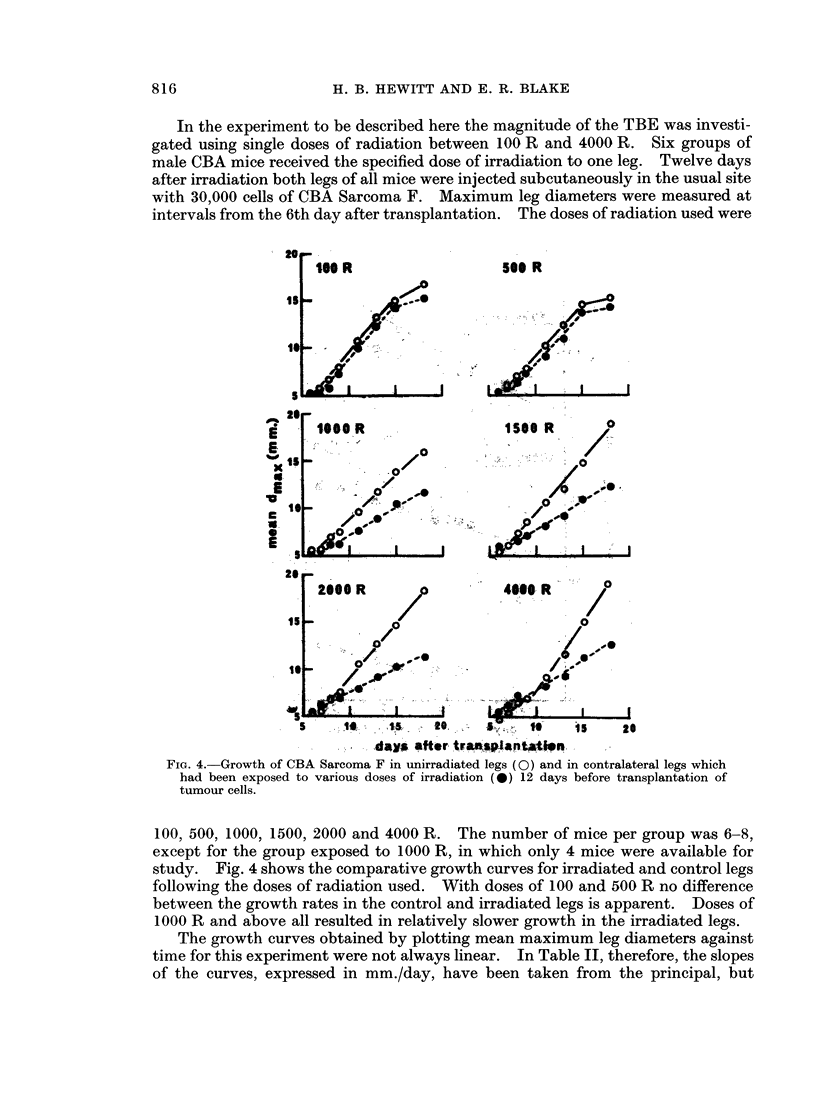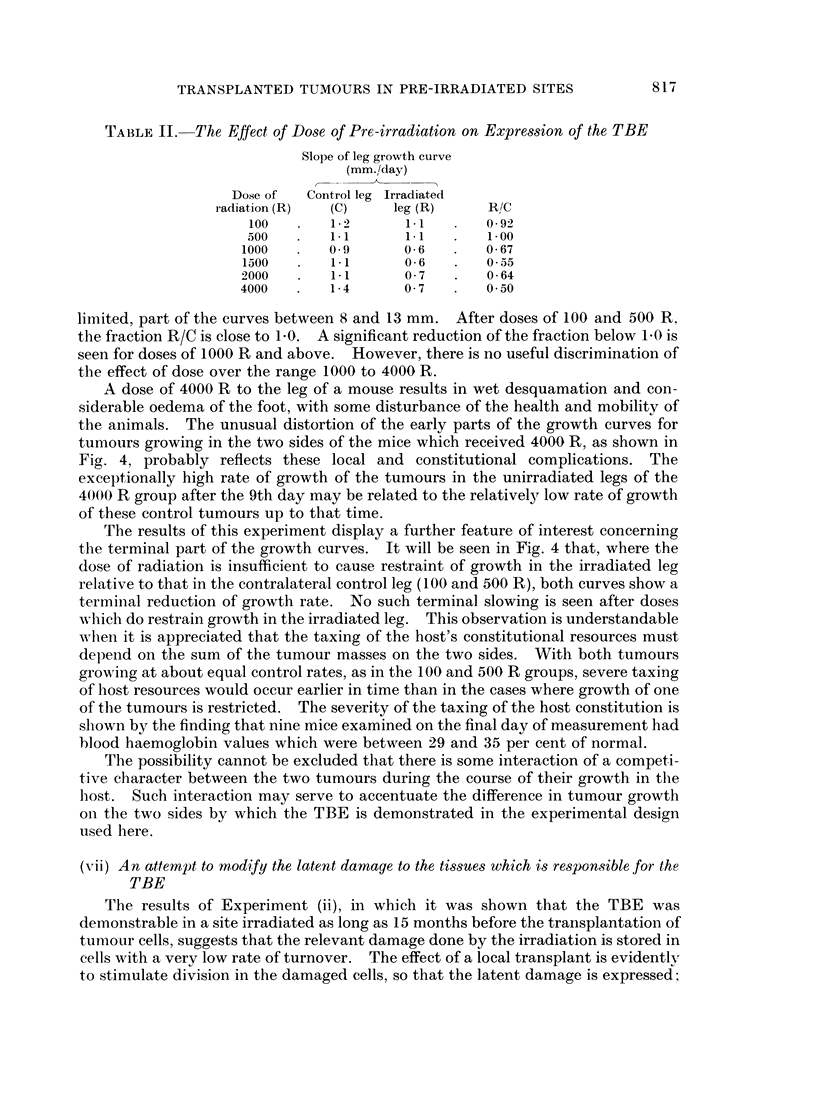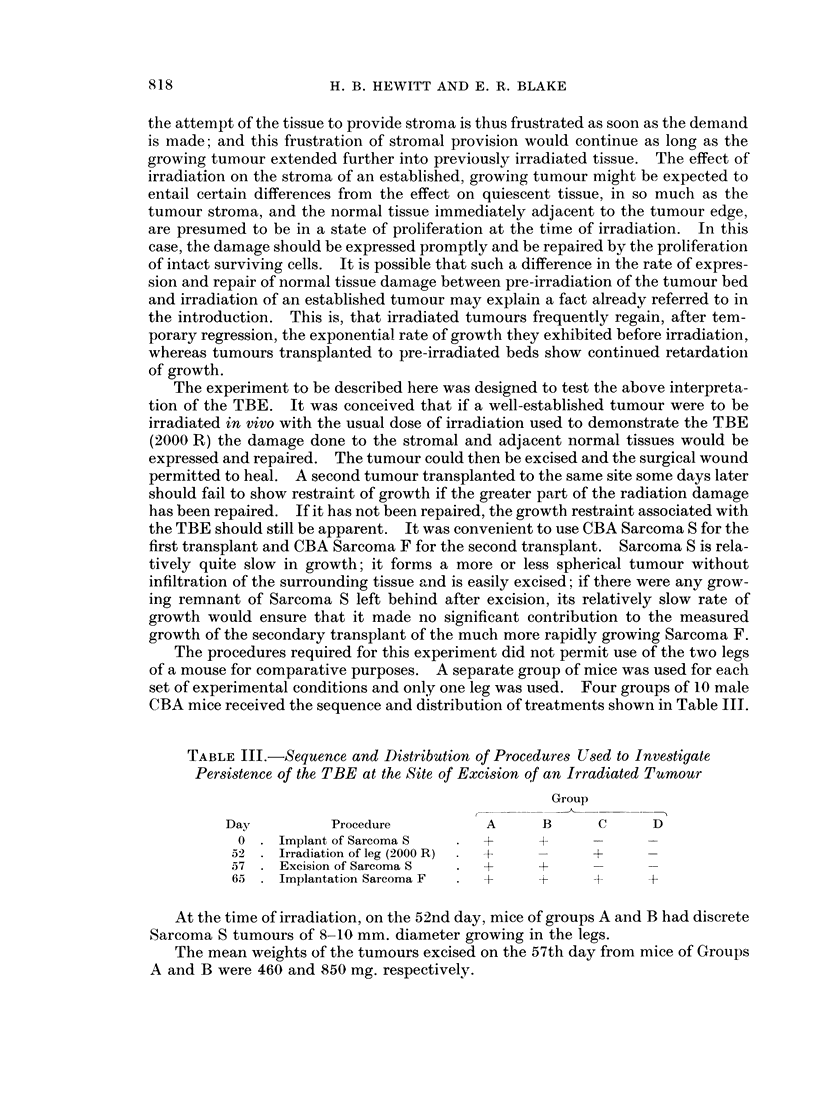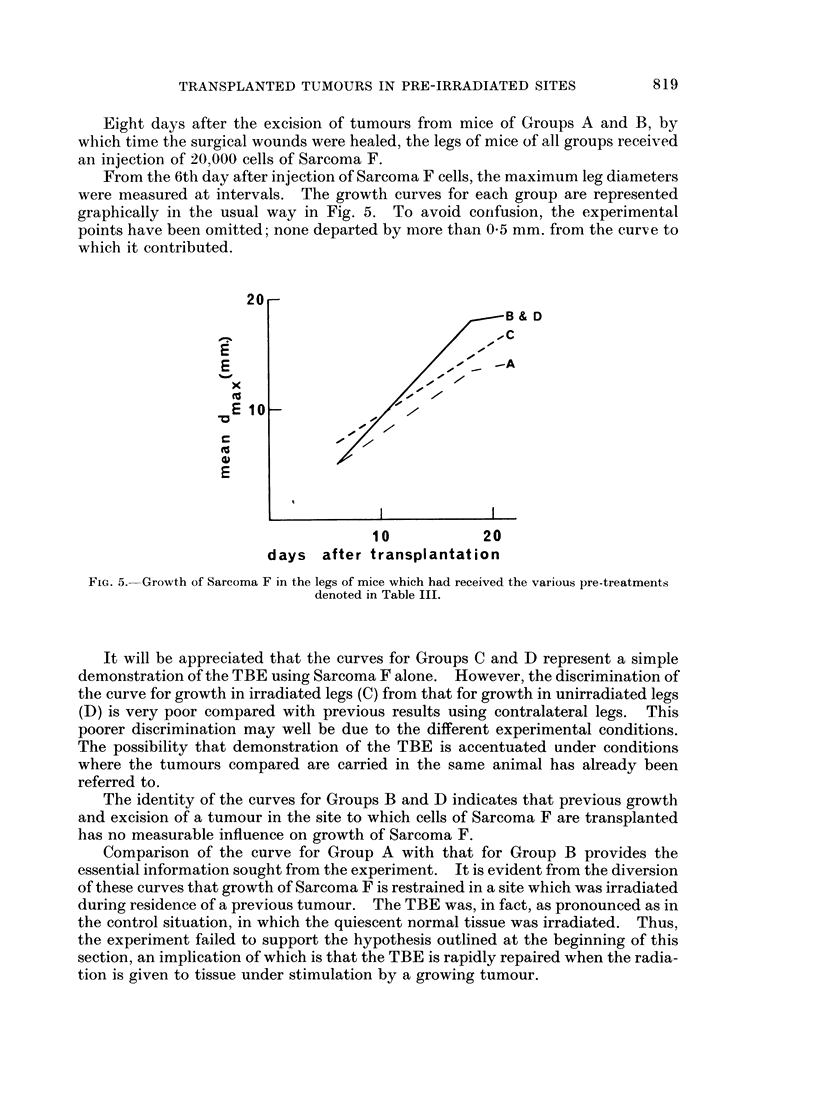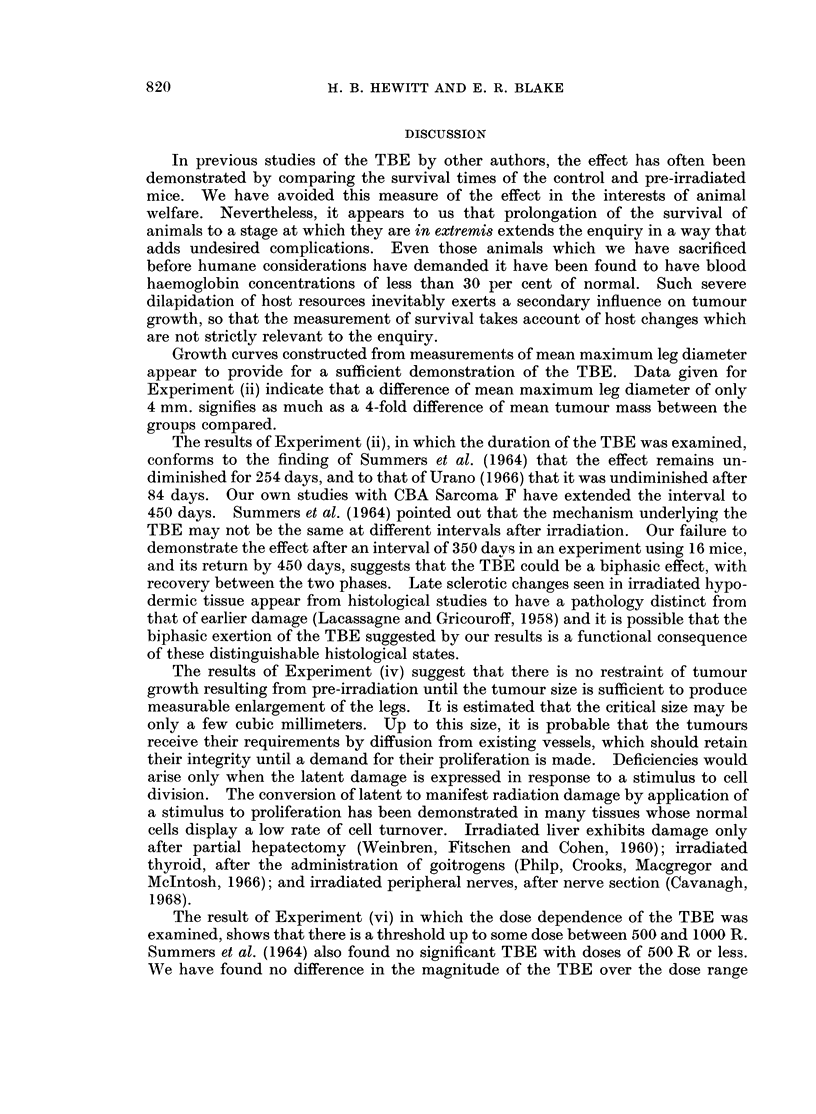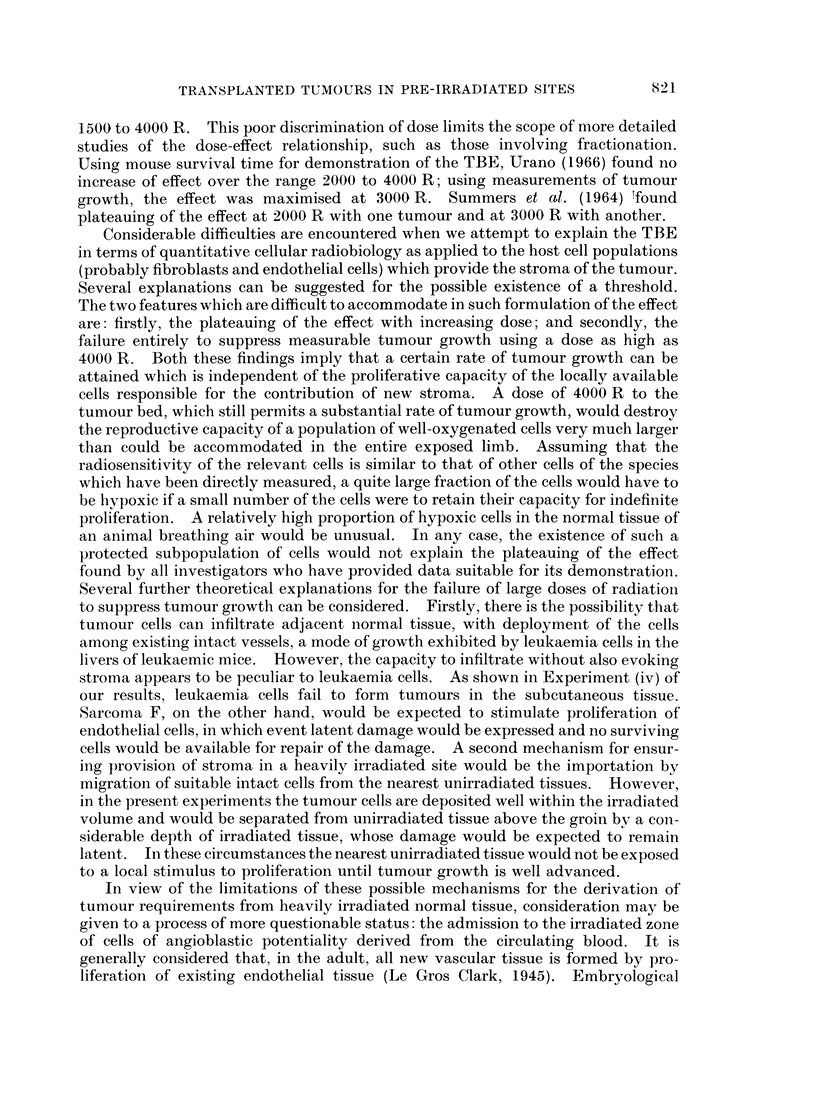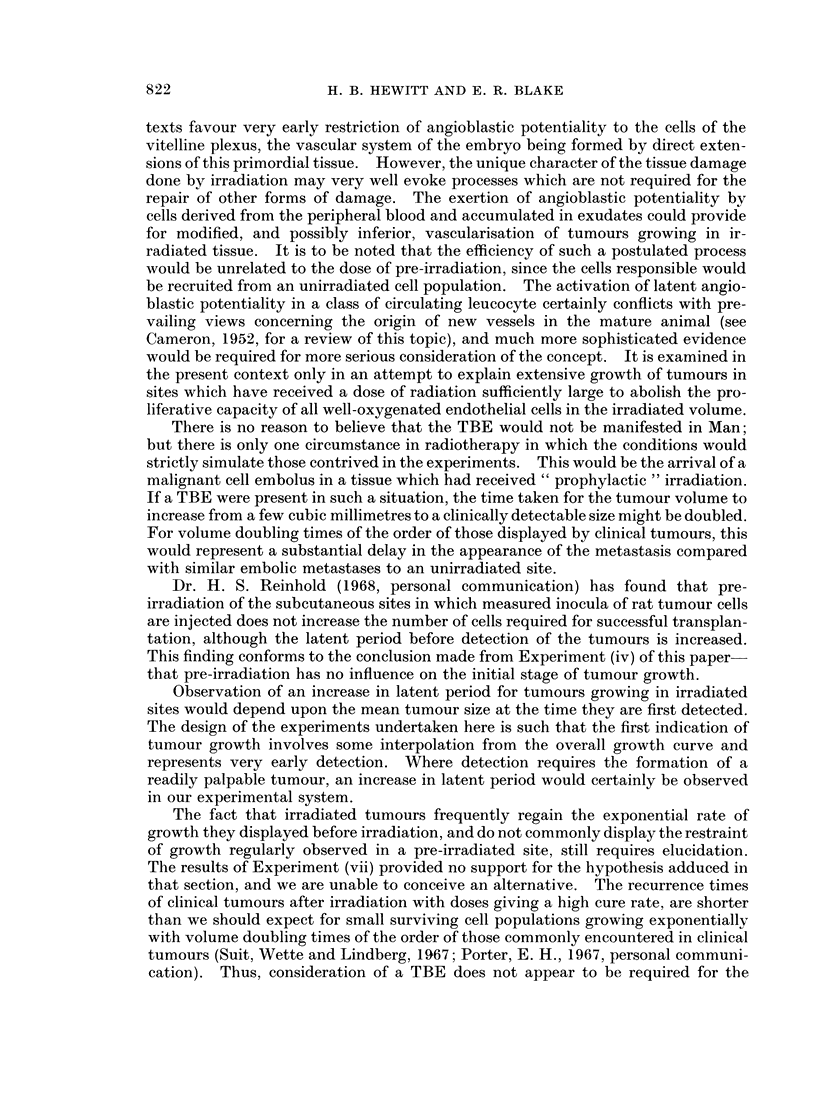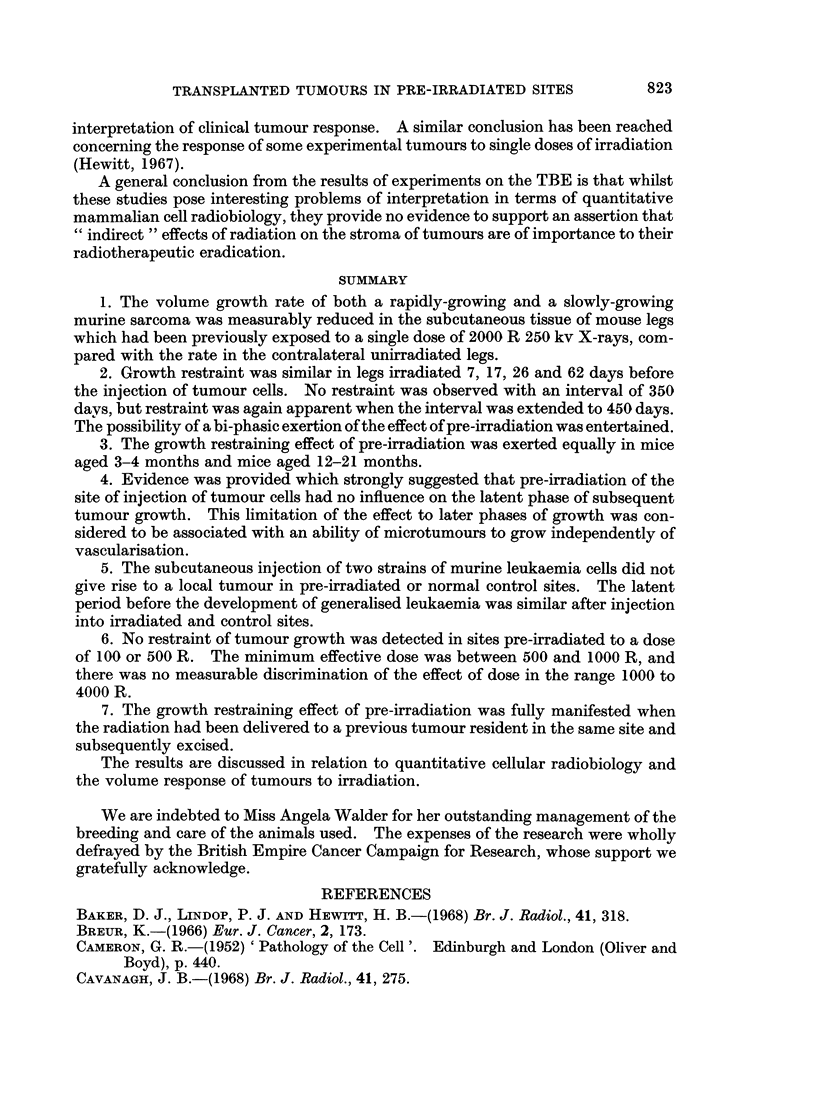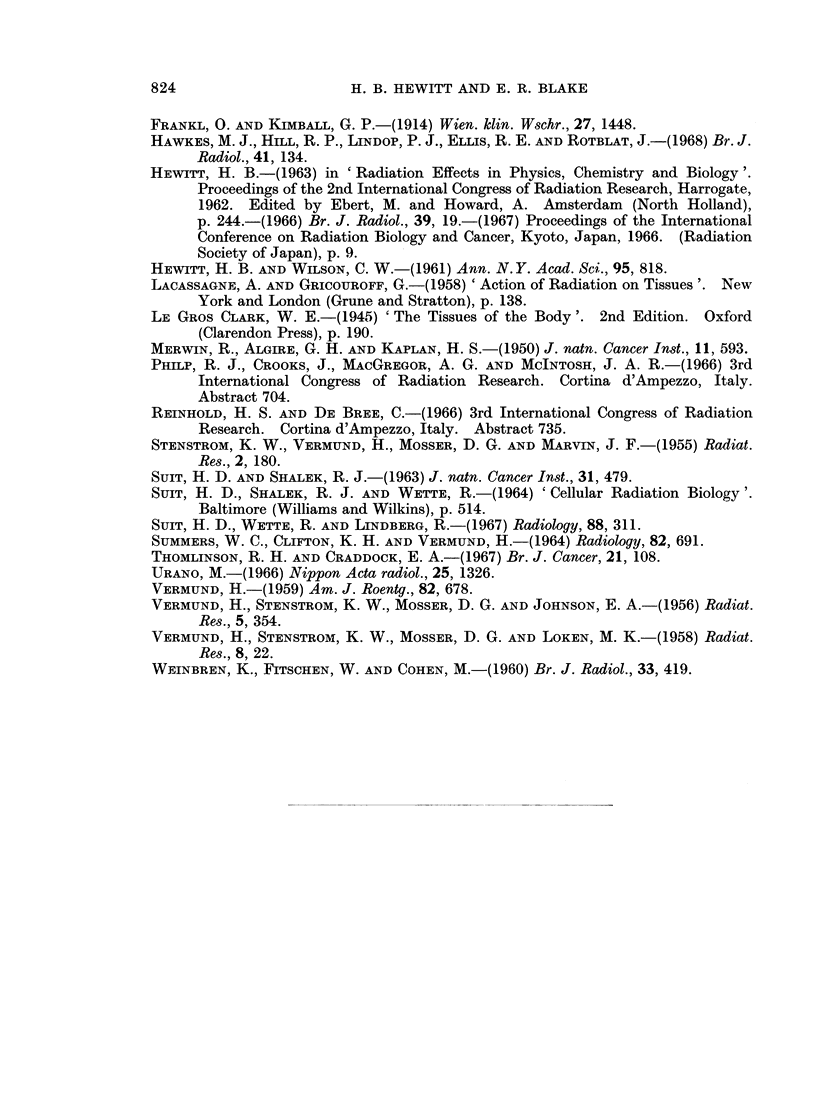# The growth of transplanted murine tumours in pre-irradiated sites.

**DOI:** 10.1038/bjc.1968.95

**Published:** 1968-12

**Authors:** H. B. Hewitt, E. R. Blake


### THE GROWTH OF TRANSPLANTED MURINE TUMOURS IN PRE-IRRADIATED SITES

H. B. HEWITT AND E. R. BLAKE

*From the British Empire Cancer Campaign Research Unit in Radiobiology, Mount Vernon Hospital, Northwood, Middlesex*

Received for publication September 3, 1968

THE obvious structural and functional interdependence of normal tissue stroma and malignant cells in solid tumours persuaded the earliest radiobiological investigators that the effect of ionising radiation on these composite structures was the resultant of separate damage to the normal and malignant tissue components. It is understandable that, with a persistent inability to quantitate separately the damage to either component, unlimited scope prevailed for the assertion of rival theories in which the response of a tumour was attributed preferentially to direct damage to one or other component. Histological study of irradiated experimental tumours is of very limited value in assessing the contribution of direct stromal damage. This follows from the fact that direct damage to blood vessels is not readily distinguishable from changes consequent on the regression of stroma which must be expected to follow the dissolution of tumour cells whose reproductive integrity has been directly damaged by the irradiation.

Recent developments in the quantitative radiobiology of mammalian tumour cells irradiated *in vivo* have encouraged interpretations of tumour response which refer, often exclusively, to the direct effect of radiation on the clonogenic cell population of the tumour. Such exclusive consideration has undoubtedly been proved to be justified in respect of the relation between the estimated size of a tumour cell population in a tumour and the single dose of radiation required for its cure under specified conditions of oxygenation. This relation has been found to accord with the predictions of relevant radiation survival curves (Hewitt, 1963; Reinhold and De Bree, 1966), this last information being obtained under conditions where stromal changes make no contribution. Suit, Shalek and Wette (1964) conclude from their extensive dose-cure studies of murine adenocarcinoma that their results " do not indicate a tissue effect on cellular radiosensitivity, tumour bed effect on tumour curability, or non-specific host-tumour effect ".

If, as it appears, radiation-induced damage to the stroma makes no measurable contribution to the eradication or " cure " of a tumour, the question remains whether such damage influences the character of the changes of tumour volume which are brought about by irradiation of a tumour *in vivo*. The consideration achieves particular importance when attempts are made to interpret tumour regrowth curves in terms of survival curves for the clonogenic tumour cells. Thomlinson and Craddock (1967) state that, for the rat fibrosarcoma they studied, the oxygen enhancement ratio determined from measurements of the growth response of their tumours to irradiation is considerably greater than that obtained from *in vitro* studies of the clonogenic cells of their tumour. They refer to capillary damage as possibly conducing to the discrepancy. Several reports have appeared of the volume response of tumours to a single or fractionated dose of radiation in which, following temporary regression, the tumours eventually revert to exponential growth at the same rate as before irradiation (Suit and Shalek, 1963; Breur, 1966; Hewitt, 1967). Although such full recovery of growth rate is not always observed (Thomlinson and Craddock, 1967; Hewitt, 1967) it is commonly assumed in the treatment of data (e.g. Hawkes, Hill, Lindop, Ellis and Rotblat, 1968) or in the development of concepts of tumour response.

Full recovery of tumour exponential growth rate following irradiation implies that radiation damage to the tumour stroma or to the normal tissue surrounding the tumour, into which it grows, does not result in restraint of growth. This apparent failure of tumour regrowth to be impaired by radiation damage to tissues by which it gains access to the constitutional resources of the host, remarkable in itself, is in conflict with the results of experiments in which tumours are transplanted into previously irradiated sites. Such experiments provide a facility for examining some features of the effect of stromal damage on tumour growth, although it must be realised that the effects of irradiation on quiescent normal tissue are unlikely to resemble closely the effects on tissue that has been stimulated by the demands of an established growing tumour.

A brief historical review by Stenstrom, Vermund, Mosser and Marvin (1955) indicates that restraint of transplanted tumour growth in previously irradiated sites, hereafter referred to as the " tumour bed effect " (TBE), has been repeatedly observed since its first demonstration by Frankl and Kimball (1914). However, much of the earlier work on the TBE was done with tumour-host systems complicated by the influence of transplantation immunity. It can be surmised that the effect of such an irrelevant influence would be greatly to exaggerate the size of the observed TBE.

Merwin, Algire and Kaplan (1950) used the Algire chamber technique to study under direct microscopic observation the fate of small solid implants of an isologous mouse tumour after transplantation to subcutaneous sites which had received 2000 or 3000 R previously. They concluded that the irradiated endothelium was rarely able to produce new vessels. On the other hand, vessels in the implant could retain function and in one case formed new vessels. Vessels also appeared in local cellular exudates; these appeared to have grown in from surrounding unirradiated tissue.

A series of papers by Vermund and his collaborators (Stenstrom *et al.*, 1955; Vermund, Stenstrom, Mosser and Johnson, 1956; Vermund, Stenstrom, Mosser and Loken, 1958; Vermund, 1959; Summers, Clifton and Vermund, 1964) describe studies of the TBE using various strains of mouse tumour injected as tumour pulp. These papers represent the most extensive recent examination of the phenomenon. These authors investigated the influence of dose of radiation and of interval between irradiation and transplantation, and compared the effect on host survival time of irradiation of tumours *in situ* and pre-irradiation of the tumour bed. Among their important observations were demonstrations that no TBE is apparent for tumours implanted intracerebrally and that host immunity is not necessarily implicated in the phenomenon. A TBE was displayed using all the tumours they studied.

Urano (1966), using a mouse sarcoma, studied the influence on the TBE of variation of dose of radiation and of interval between irradiation and transplantation. The potentiality of tumour growth was measured in terms of growth curves and host survival time.

The present paper describes further investigations of the TBE using two murine tumours for which other radiobiological data have been reported previously. It was desired to examine the applicability to these tumours of some of the principal findings of previous investigators and to extend the enquiry along lines that are of interest in the context of our current investigations. We have studied the duration of the TBE and its persistence at the site of an excised irradiated tumour, the phase of tumour growth at which the influence operates, the relation between radiation dose and the magnitude of the TBE, and some other features of the phenomenon.

### MATERIALS AND METHODS

*Mice*

Mice of both sexes of inbred strain CBA/Ht were used at 2–4 months of age unless otherwise stated.

*Tumours*

Both solid tumours employed arose spontaneously in the same inbred colony of mice as was used for the experiments. They were maintained by serial subcutaneous passage in isologous mice. In extensive studies undertaken before and during the present experiments neither has shown evidence of significant antigenicity. The following tumours were used:

(a) *CBA/Ht Sarcoma F.*—This is an anaplastic sarcoma which grows with a volume doubling time of about 2 days. The tumour yields single-cell suspensions of viable cells on digestion of minced tumour with pancreatic enzymes, and has been used in radiobiological studies reported previously (Hewitt and Wilson, 1961; Hewitt, 1966; Baker, Lindop and Hewitt, 1968).

(b) *CBA/Ht Sarcoma S.*—This sarcoma has been transplanted serially for several years, during which the volume doubling time has remained at about 10 days, an exceptionally slow rate of growth for a long transplanted tumour. No success has been had with attempts to produce single-cell suspensions from this tumour, which was transplanted by the surgical implantation of tumour fragments of a few $mm.^3$.

(c) *CBA/Ht Leukaemias S and R–I.*—Of these two strains, S arose spontaneously, and R–I was radiation-induced, in mice of the substrain which provided the mice used in the experiments. In each case, transplantation to 50 per cent of injected mice can be effected with about 2 cells. Neither strain gives rise to ascites after intraperitoneal injection and both strains produce a final leukaemic state characterised by gross infiltration of the liver and spleen.

*Irradiation*

Mice to be irradiated were sedated with 0·125 ml./20 g. body weight of a 2·5 per cent solution in saline of tribromoethanol (" Avertin ", Winthrop Laboratories, N.Y.) injected subcutaneously. They were placed in individual lead boxes from which one leg was retained out of the box up to the groin by sticky tape applied to the foot. Mice were exposed locally in groups of eight at a time to 250 kv X-rays generated at 15 mA and filtered through 0·5 mm. Cu and 1·0 mm. Al. The dose rate, measured with small condenser ionisation chambers located in the site of a leg, was 360 R/min. Except where stated otherwise, a single dose of 2000 R was delivered to legs of mice breathing air during irradiation. This dose produced epilation and slight temporary oedema of exposed legs, but no ulceration.

*Transplantation of tumours*

CBA Sarcoma F was transplanted to the legs of mice using counted single-cell suspensions prepared by a digestion technique described previously (Hewitt, 1966). A volume of 0·05 ml. of suspension was injected through a 22-gauge hypodermic needle into the lowest lateral part of the leg just above the ankle joint. The number of tumour cells injected was usually 20,000, representing about 1000 times the number of cells required to give 50 per cent of takes.

CBA Sarcoma S was transplanted as cubes of a few mm.$^3$ of healthy tumour inserted subcutaneously into the lower leg laterally through a small transverse incision which was closed with a metal skin clip.

All experiments were performed aseptically, and the injections and surgical procedures were all performed under ether anaesthesia.

An experimental group usually comprised 8 mice—4 with the left leg irradiated and 4 with the right. In most experiments, each animal received a transplant in the irradiated and unirradiated leg.

*Measurement of tumour growth*

The maximum diameter of each leg was measured with calipers every 1 or 2 days from about the 6th day after transplantation until humane considerations required killing of the mice. The maximum diameter of normal legs was 4·5–5·5 mm., and tumour-bearing mice were killed when either tumour attained a diameter of 15–20 mm. Mean maximum leg diameters in mm. were plotted against days after transplantation. A straight line could usually be well fitted to the points by eye. Growth curves for the tumours growing in irradiated and control legs were plotted together, the TBE being denoted by diversion of the curves. The measurements made did not permit an estimate of tumour mass to be made. However, their adequacy for comparative purposes was justified by the absence of any difference of tumour shape on the two sides. In a few experiments, following the final measurements, tumours were excised and weighed. A 1 : 4 ratio of mean tumour weights was obtained when the mean maximum leg diameters were 12 and 16 mm. respectively.

### EXPERIMENTS AND RESULTS

(i) *The influence of intrinsic tumour growth rate on expression of the TBE*

Fig. 1 compares the TBE as demonstrated using the rapidly growing CBA Sarcoma F (A) and the slowly growing CBA Sarcoma S (B). The ratios of the slopes of the curves for the control and irradiated legs are 1 : 0·6 and 1 : 0·42 respectively. Since the range of growth rates covered by these two tumours would include a high proportion of all murine tumours, it is concluded that intrinsic tumour growth rate has little or no influence on expression of the TBE. It is of some interest that the magnitude of the effect is not reduced when the rate of demand for stroma is reduced.

(ii) *Persistence of the TBE*

Six groups each of 8 CBA female mice received 2000 R to 1 leg. At intervals of 7, 17, 24, 62, 350 or 450 days after irradiation a group received an injection into both legs of 20,000 CBA Sarcoma F cells. Growth curves were constructed from serial measurements of the legs in the usual way. In all cases a straight line could be well fitted to the points by eye. The results of the experiment are shown in

Fig. 1.—Growth of CBA tumours in unirradiated legs (○) and in contralateral legs which received 2000 R X-rays before transplantation of tumour (●). A—rapidly-growing Sarcoma F; B—slowly-growing Sarcoma S.

Table I.—*The Effect of Length of Interval between Irradiation and Transplantation of Tumour Cells on Expression of the TBE*

| Interval (days) | Slope of leg growth curve (mm./day): Control leg (C) | Irradiated leg (R) | R/C |
|---|---|---|---|
| 7 | 1·5 | 0·7 | 0·47 |
| 17 | 1·3 | 0·8 | 0·62 |
| 24 | 1·3 | 0·7 | 0·54 |
| 62 | 1·3 | 0·7 | 0·54 |
| 350 | 0·9 | 0·9 | 1·00 |
| 450 | 1·0 | 0·5 | 0·50 |

Table I, in which the slopes of the two curves for each group are given in terms of mm./day. In the final vertical column, the slope of the curve for irradiated legs is given as a fraction of the slope of the corresponding curve for the unirradiated legs. The rate of growth of tumour in the control legs for intervals 350 and 450 days is distinctly less than for shorter intervals. The result of the experiment which follows (iii) shows that this fall in control tumour growth rate is not due to the greater age, at the time of transplantation, of the mice used for the longer intervals. A review of growth data for Sarcoma F over the period occupied by the present experiment suggested that there had been a spontaneous alteration of the growth potential of the tumour within the tumour-host system used.

In all groups except that for an interval of 350 days, the growth rate of irradiated legs was only about half that in the corresponding control legs. The group for a 350-day interval is distinctive in that the rate of tumour growth in the irradiated legs is indistinguishable from that in the control legs. Owing to loss of mice in the later stages of the experiment, only 5 mice were available for the final group (450-day interval). In this group, however, the data were augmented by excising and weighing all tumours after the final leg measurements on the 19th day after transplantation. The tumours from the control legs weighed 2·0–3·1 g. (mean 2·4 g.; S.D. 0·38); those from the legs irradiated 450 days previously weighed 0·6–0·9 g. (mean 0·64 g.; S.D. 0·28). Thus, there is substantial evidence from these results that radiation-induced damage to the subcutaneous tissue which is sufficient to restrain the growth of tumours transplanted to the irradiated site persists throughout a large part of a mouse's lifetime.

The failure to demonstrate restraint of growth in the legs irradiated 350 days previously deserves attention because it is the only failure to demonstrate a TBE in all the numerous experiments we have done using this tumour and a dose of radiation of 2000 R. It should be added that 16 mice, twice the usual number, contributed the data for this interval.

Owing to the time scale of the experiment, it has not yet been possible to re-examine the TBE in the region of this interval of time between irradiation and transplantation, to confirm that the TBE is exerted in two phases separated by an interval of recovery. Such a bi-phasic effect of irradiation is a well-known phenomenon in clinical radiotherapy, where earlier and later effects of radiation on the normal tissues represent entities of different pathology.

(iii) *The effect of age of host on expression of the TBE*

The conditions of the last experiment (ii) involved a wide difference in the ages of the mice of different groups at the time tumour cells were injected. It was, therefore, desirable to ensure that the TBE is equally expressed in relatively young and old mice. In Fig. 2 are shown the growth curves for control and irradiated legs using female CBA mice of age groups 96–117 days and 390–635 days. The comparison was made in a single experiment in which the interval between irradiation and transplantation was 20 days. The figure shows that the TBE is equally expressed in mice of the two age groups and that host age has not influenced the rate of growth of the tumours in either control or irradiated legs. It is concluded that the results of the last experiment were not complicated by differences of the age of the mice at the time of transplantation.

(iv) *Phase of tumour growth during which TBE influences growth*

It has been observed that in all cases where mean maximum leg diameter growth curves for control and irradiated legs have been charted together the two linear curves intersect at a point corresponding to the maximum diameter of the normal mouse leg (about 5 mm.). That is, measurable enlargement of the legs occurs at about the same time on the two sides; the latent periods of growth appear to be similar. In the case of tumours grown from relatively small inocula of tumour cells and in which a latent period of 6–8 days is observed before measurable growth is apparent, the absence of a difference of latent period for tumours grown in the control and irradiated legs implies that no relative restriction of growth occurs in the irradiated legs during this early phase of growth. The following experiment was designed to enhance the opportunity of detecting a difference of latent period for tumours growing in control and irradiated sites.

Three groups of 8 male CBA mice received 2000 R to one leg. Thirteen days after irradiation, mice of the three groups received in both legs an injection of 78,000, 10,000, or 1300 cells of CBA Sarcoma F. Maximum leg diameters were measured at intervals after injection of tumour cells, and growth curves were plotted in the usual way. Fig. 3 shows that the TBE was equally apparent in all three pairs of curves. In all groups, the linear growth curves for control and irradiated legs intersect at a point corresponding to a normal leg diameter of about 5 mm. on the ordinate. As expected, the point of intersection is displaced progressively to the right as the size of the inoculum is reduced. Even with the lowest cell dose used, however, the latent period of tumours on the irradiated side

FIG. 2.—Growth of Sarcoma F in unirradiated (△) and pre-irradiated (▲) legs of young mice, and in unirradiated (○) and pre-irradiated (●) legs of old mice. Exposure dose of irradiation, 2000 R. Time between irradiation and tumour transplantation, 20 days.

cannot be distinguished from that of tumours on the control side. If the relative restriction of growth in the irradiated leg, as seen during the measurable phase of growth, were being exerted during the occult phase of growth, a difference of latent period should be observed for tumours growing on the two sides, and this difference should increase as the size of the inoculum is reduced. The evidence provided by this experiment strongly suggests that restraint of tumour growth in an irradiated site is not exerted until the tumour approaches a size of at least 1 mm.[3]. This finding is not unexpected when it is appreciated that microtumours can obtain their nutritional requirements by diffusion from existing normal vessels, and would suffer relative nutritional deprivation only when they attained a size requiring their vascularisation. The incapacity of the irradiated site to provide new stroma by the proliferation of resident endothelial elements would only become apparent after the demand is made.

(v) *The TBE in relation to leukaemia*

The TBE was sought in two separate experiments using different leukaemia strains. In each experiment, a constant inoculum of leukaemia cells was injected into the irradiated legs of 8 mice and into the unirradiated legs of an equivalent group. None of the injected mice developed a tumour at the site of injection before the animals became moribund with the generalised disease. In the case of both leukaemia strains, there was no significant difference in mean latent periods before development of generalised disease in the two groups of mice compared.

FIG. 3.—Growth of Sarcoma F in unirradiated (○) and contralateral irradiated (●) legs. Effect of varying size of inoculum of tumour cells. Exposure dose of irradiation, 2000 R; interval between irradiation and transplantation, 13 days.

Since no local tumour is formed before dissemination of the disease, the injected cells evidently migrate to the viscera along existing tissue spaces, lymphatics or blood vessels. No stroma is called for, and therefore no delay is to be expected in the development of the generalised disease after injection into irradiated sites.

(vi) *The effect of dose of irradiation on the magnitude of the TBE*

The standard single dose of irradiation used in most of the experiments in this investigation of the TBE is 2000 R. This dose was found to be sufficient for regular demonstration of the TBE yet below the necrotising dose for mouse skin.

In the experiment to be described here the magnitude of the TBE was investigated using single doses of radiation between 100 R and 4000 R. Six groups of male CBA mice received the specified dose of irradiation to one leg. Twelve days after irradiation both legs of all mice were injected subcutaneously in the usual site with 30,000 cells of CBA Sarcoma F. Maximum leg diameters were measured at intervals from the 6th day after transplantation. The doses of radiation used were

FIG. 4.—Growth of CBA Sarcoma F in unirradiated legs (○) and in contralateral legs which had been exposed to various doses of irradiation (●) 12 days before transplantation of tumour cells.

100, 500, 1000, 1500, 2000 and 4000 R. The number of mice per group was 6–8, except for the group exposed to 1000 R, in which only 4 mice were available for study. Fig. 4 shows the comparative growth curves for irradiated and control legs following the doses of radiation used. With doses of 100 and 500 R no difference between the growth rates in the control and irradiated legs is apparent. Doses of 1000 R and above all resulted in relatively slower growth in the irradiated legs.

The growth curves obtained by plotting mean maximum leg diameters against time for this experiment were not always linear. In Table II, therefore, the slopes of the curves, expressed in mm./day, have been taken from the principal, but

TABLE II.—*The Effect of Dose of Pre-irradiation on Expression of the TBE*

| Dose of radiation (R) | Slope of leg growth curve (mm./day) Control leg (C) | Irradiated leg (R) | R/C |
|---|---|---|---|
| 100 | 1·2 | 1·1 | 0·92 |
| 500 | 1·1 | 1·1 | 1·00 |
| 1000 | 0·9 | 0·6 | 0·67 |
| 1500 | 1·1 | 0·6 | 0·55 |
| 2000 | 1·1 | 0·7 | 0·64 |
| 4000 | 1·4 | 0·7 | 0·50 |

limited, part of the curves between 8 and 13 mm. After doses of 100 and 500 R, the fraction R/C is close to 1·0. A significant reduction of the fraction below 1·0 is seen for doses of 1000 R and above. However, there is no useful discrimination of the effect of dose over the range 1000 to 4000 R.

A dose of 4000 R to the leg of a mouse results in wet desquamation and considerable oedema of the foot, with some disturbance of the health and mobility of the animals. The unusual distortion of the early parts of the growth curves for tumours growing in the two sides of the mice which received 4000 R, as shown in Fig. 4, probably reflects these local and constitutional complications. The exceptionally high rate of growth of the tumours in the unirradiated legs of the 4000 R group after the 9th day may be related to the relatively low rate of growth of these control tumours up to that time.

The results of this experiment display a further feature of interest concerning the terminal part of the growth curves. It will be seen in Fig. 4 that, where the dose of radiation is insufficient to cause restraint of growth in the irradiated leg relative to that in the contralateral control leg (100 and 500 R), both curves show a terminal reduction of growth rate. No such terminal slowing is seen after doses which do restrain growth in the irradiated leg. This observation is understandable when it is appreciated that the taxing of the host's constitutional resources must depend on the sum of the tumour masses on the two sides. With both tumours growing at about equal control rates, as in the 100 and 500 R groups, severe taxing of host resources would occur earlier in time than in the cases where growth of one of the tumours is restricted. The severity of the taxing of the host constitution is shown by the finding that nine mice examined on the final day of measurement had blood haemoglobin values which were between 29 and 35 per cent of normal.

The possibility cannot be excluded that there is some interaction of a competitive character between the two tumours during the course of their growth in the host. Such interaction may serve to accentuate the difference in tumour growth on the two sides by which the TBE is demonstrated in the experimental design used here.

(vii) *An attempt to modify the latent damage to the tissues which is responsible for the TBE*

The results of Experiment (ii), in which it was shown that the TBE was demonstrable in a site irradiated as long as 15 months before the transplantation of tumour cells, suggests that the relevant damage done by the irradiation is stored in cells with a very low rate of turnover. The effect of a local transplant is evidently to stimulate division in the damaged cells, so that the latent damage is expressed; the attempt of the tissue to provide stroma is thus frustrated as soon as the demand is made; and this frustration of stromal provision would continue as long as the growing tumour extended further into previously irradiated tissue. The effect of irradiation on the stroma of an established, growing tumour might be expected to entail certain differences from the effect on quiescent tissue, in so much as the tumour stroma, and the normal tissue immediately adjacent to the tumour edge, are presumed to be in a state of proliferation at the time of irradiation. In this case, the damage should be expressed promptly and be repaired by the proliferation of intact surviving cells. It is possible that such a difference in the rate of expression and repair of normal tissue damage between pre-irradiation of the tumour bed and irradiation of an established tumour may explain a fact already referred to in the introduction. This is, that irradiated tumours frequently regain, after temporary regression, the exponential rate of growth they exhibited before irradiation, whereas tumours transplanted to pre-irradiated beds show continued retardation of growth.

The experiment to be described here was designed to test the above interpretation of the TBE. It was conceived that if a well-established tumour were to be irradiated *in vivo* with the usual dose of irradiation used to demonstrate the TBE (2000 R) the damage done to the stromal and adjacent normal tissues would be expressed and repaired. The tumour could then be excised and the surgical wound permitted to heal. A second tumour transplanted to the same site some days later should fail to show restraint of growth if the greater part of the radiation damage has been repaired. If it has not been repaired, the growth restraint associated with the TBE should still be apparent. It was convenient to use CBA Sarcoma S for the first transplant and CBA Sarcoma F for the second transplant. Sarcoma S is relatively quite slow in growth; it forms a more or less spherical tumour without infiltration of the surrounding tissue and is easily excised; if there were any growing remnant of Sarcoma S left behind after excision, its relatively slow rate of growth would ensure that it made no significant contribution to the measured growth of the secondary transplant of the much more rapidly growing Sarcoma F.

The procedures required for this experiment did not permit use of the two legs of a mouse for comparative purposes. A separate group of mice was used for each set of experimental conditions and only one leg was used. Four groups of 10 male CBA mice received the sequence and distribution of treatments shown in Table III.

TABLE III.—*Sequence and Distribution of Procedures Used to Investigate Persistence of the TBE at the Site of Excision of an Irradiated Tumour*

| | | Group | | | |
|---|---|---|---|---|---|
| Day | Procedure | A | B | C | D |
| 0 | Implant of Sarcoma S | + | + | — | — |
| 52 | Irradiation of leg (2000 R) | + | — | + | — |
| 57 | Excision of Sarcoma S | + | + | — | — |
| 65 | Implantation Sarcoma F | + | + | + | + |

At the time of irradiation, on the 52nd day, mice of groups A and B had discrete Sarcoma S tumours of 8–10 mm. diameter growing in the legs.

The mean weights of the tumours excised on the 57th day from mice of Groups A and B were 460 and 850 mg. respectively.

Eight days after the excision of tumours from mice of Groups A and B, by which time the surgical wounds were healed, the legs of mice of all groups received an injection of 20,000 cells of Sarcoma F.

From the 6th day after injection of Sarcoma F cells, the maximum leg diameters were measured at intervals. The growth curves for each group are represented graphically in the usual way in Fig. 5. To avoid confusion, the experimental points have been omitted; none departed by more than 0·5 mm. from the curve to which it contributed.

Fig. 5.—Growth of Sarcoma F in the legs of mice which had received the various pre-treatments denoted in Table III.

It will be appreciated that the curves for Groups C and D represent a simple demonstration of the TBE using Sarcoma F alone. However, the discrimination of the curve for growth in irradiated legs (C) from that for growth in unirradiated legs (D) is very poor compared with previous results using contralateral legs. This poorer discrimination may well be due to the different experimental conditions. The possibility that demonstration of the TBE is accentuated under conditions where the tumours compared are carried in the same animal has already been referred to.

The identity of the curves for Groups B and D indicates that previous growth and excision of a tumour in the site to which cells of Sarcoma F are transplanted has no measurable influence on growth of Sarcoma F.

Comparison of the curve for Group A with that for Group B provides the essential information sought from the experiment. It is evident from the diversion of these curves that growth of Sarcoma F is restrained in a site which was irradiated during residence of a previous tumour. The TBE was, in fact, as pronounced as in the control situation, in which the quiescent normal tissue was irradiated. Thus, the experiment failed to support the hypothesis outlined at the beginning of this section, an implication of which is that the TBE is rapidly repaired when the radiation is given to tissue under stimulation by a growing tumour.

### DISCUSSION

In previous studies of the TBE by other authors, the effect has often been demonstrated by comparing the survival times of the control and pre-irradiated mice. We have avoided this measure of the effect in the interests of animal welfare. Nevertheless, it appears to us that prolongation of the survival of animals to a stage at which they are *in extremis* extends the enquiry in a way that adds undesired complications. Even those animals which we have sacrificed before humane considerations have demanded it have been found to have blood haemoglobin concentrations of less than 30 per cent of normal. Such severe dilapidation of host resources inevitably exerts a secondary influence on tumour growth, so that the measurement of survival takes account of host changes which are not strictly relevant to the enquiry.

Growth curves constructed from measurements of mean maximum leg diameter appear to provide for a sufficient demonstration of the TBE. Data given for Experiment (ii) indicate that a difference of mean maximum leg diameter of only 4 mm. signifies as much as a 4-fold difference of mean tumour mass between the groups compared.

The results of Experiment (ii), in which the duration of the TBE was examined, conforms to the finding of Summers *et al.* (1964) that the effect remains undiminished for 254 days, and to that of Urano (1966) that it was undiminished after 84 days. Our own studies with CBA Sarcoma F have extended the interval to 450 days. Summers *et al.* (1964) pointed out that the mechanism underlying the TBE may not be the same at different intervals after irradiation. Our failure to demonstrate the effect after an interval of 350 days in an experiment using 16 mice, and its return by 450 days, suggests that the TBE could be a biphasic effect, with recovery between the two phases. Late sclerotic changes seen in irradiated hypodermic tissue appear from histological studies to have a pathology distinct from that of earlier damage (Lacassagne and Gricouroff, 1958) and it is possible that the biphasic exertion of the TBE suggested by our results is a functional consequence of these distinguishable histological states.

The results of Experiment (iv) suggest that there is no restraint of tumour growth resulting from pre-irradiation until the tumour size is sufficient to produce measurable enlargement of the legs. It is estimated that the critical size may be only a few cubic millimeters. Up to this size, it is probable that the tumours receive their requirements by diffusion from existing vessels, which should retain their integrity until a demand for their proliferation is made. Deficiencies would arise only when the latent damage is expressed in response to a stimulus to cell division. The conversion of latent to manifest radiation damage by application of a stimulus to proliferation has been demonstrated in many tissues whose normal cells display a low rate of cell turnover. Irradiated liver exhibits damage only after partial hepatectomy (Weinbren, Fitschen and Cohen, 1960); irradiated thyroid, after the administration of goitrogens (Philp, Crooks, Macgregor and McIntosh, 1966); and irradiated peripheral nerves, after nerve section (Cavanagh, 1968).

The result of Experiment (vi) in which the dose dependence of the TBE was examined, shows that there is a threshold up to some dose between 500 and 1000 R. Summers *et al.* (1964) also found no significant TBE with doses of 500 R or less. We have found no difference in the magnitude of the TBE over the dose range

1500 to 4000 R. This poor discrimination of dose limits the scope of more detailed studies of the dose-effect relationship, such as those involving fractionation. Using mouse survival time for demonstration of the TBE, Urano (1966) found no increase of effect over the range 2000 to 4000 R; using measurements of tumour growth, the effect was maximised at 3000 R. Summers *et al.* (1964) found plateauing of the effect at 2000 R with one tumour and at 3000 R with another.

Considerable difficulties are encountered when we attempt to explain the TBE in terms of quantitative cellular radiobiology as applied to the host cell populations (probably fibroblasts and endothelial cells) which provide the stroma of the tumour. Several explanations can be suggested for the possible existence of a threshold. The two features which are difficult to accommodate in such formulation of the effect are: firstly, the plateauing of the effect with increasing dose; and secondly, the failure entirely to suppress measurable tumour growth using a dose as high as 4000 R. Both these findings imply that a certain rate of tumour growth can be attained which is independent of the proliferative capacity of the locally available cells responsible for the contribution of new stroma. A dose of 4000 R to the tumour bed, which still permits a substantial rate of tumour growth, would destroy the reproductive capacity of a population of well-oxygenated cells very much larger than could be accommodated in the entire exposed limb. Assuming that the radiosensitivity of the relevant cells is similar to that of other cells of the species which have been directly measured, a quite large fraction of the cells would have to be hypoxic if a small number of the cells were to retain their capacity for indefinite proliferation. A relatively high proportion of hypoxic cells in the normal tissue of an animal breathing air would be unusual. In any case, the existence of such a protected subpopulation of cells would not explain the plateauing of the effect found by all investigators who have provided data suitable for its demonstration. Several further theoretical explanations for the failure of large doses of radiation to suppress tumour growth can be considered. Firstly, there is the possibility that tumour cells can infiltrate adjacent normal tissue, with deployment of the cells among existing intact vessels, a mode of growth exhibited by leukaemia cells in the livers of leukaemic mice. However, the capacity to infiltrate without also evoking stroma appears to be peculiar to leukaemia cells. As shown in Experiment (iv) of our results, leukaemia cells fail to form tumours in the subcutaneous tissue. Sarcoma F, on the other hand, would be expected to stimulate proliferation of endothelial cells, in which event latent damage would be expressed and no surviving cells would be available for repair of the damage. A second mechanism for ensuring provision of stroma in a heavily irradiated site would be the importation by migration of suitable intact cells from the nearest unirradiated tissues. However, in the present experiments the tumour cells are deposited well within the irradiated volume and would be separated from unirradiated tissue above the groin by a considerable depth of irradiated tissue, whose damage would be expected to remain latent. In these circumstances the nearest unirradiated tissue would not be exposed to a local stimulus to proliferation until tumour growth is well advanced.

In view of the limitations of these possible mechanisms for the derivation of tumour requirements from heavily irradiated normal tissue, consideration may be given to a process of more questionable status: the admission to the irradiated zone of cells of angioblastic potentiality derived from the circulating blood. It is generally considered that, in the adult, all new vascular tissue is formed by proliferation of existing endothelial tissue (Le Gros Clark, 1945). Embryological texts favour very early restriction of angioblastic potentiality to the cells of the vitelline plexus, the vascular system of the embryo being formed by direct extensions of this primordial tissue. However, the unique character of the tissue damage done by irradiation may very well evoke processes which are not required for the repair of other forms of damage. The exertion of angioblastic potentiality by cells derived from the peripheral blood and accumulated in exudates could provide for modified, and possibly inferior, vascularisation of tumours growing in irradiated tissue. It is to be noted that the efficiency of such a postulated process would be unrelated to the dose of pre-irradiation, since the cells responsible would be recruited from an unirradiated cell population. The activation of latent angioblastic potentiality in a class of circulating leucocyte certainly conflicts with prevailing views concerning the origin of new vessels in the mature animal (see Cameron, 1952, for a review of this topic), and much more sophisticated evidence would be required for more serious consideration of the concept. It is examined in the present context only in an attempt to explain extensive growth of tumours in sites which have received a dose of radiation sufficiently large to abolish the proliferative capacity of all well-oxygenated endothelial cells in the irradiated volume.

There is no reason to believe that the TBE would not be manifested in Man; but there is only one circumstance in radiotherapy in which the conditions would strictly simulate those contrived in the experiments. This would be the arrival of a malignant cell embolus in a tissue which had received " prophylactic " irradiation. If a TBE were present in such a situation, the time taken for the tumour volume to increase from a few cubic millimetres to a clinically detectable size might be doubled. For volume doubling times of the order of those displayed by clinical tumours, this would represent a substantial delay in the appearance of the metastasis compared with similar embolic metastases to an unirradiated site.

Dr. H. S. Reinhold (1968, personal communication) has found that pre-irradiation of the subcutaneous sites in which measured inocula of rat tumour cells are injected does not increase the number of cells required for successful transplantation, although the latent period before detection of the tumours is increased. This finding conforms to the conclusion made from Experiment (iv) of this paper—that pre-irradiation has no influence on the initial stage of tumour growth.

Observation of an increase in latent period for tumours growing in irradiated sites would depend upon the mean tumour size at the time they are first detected. The design of the experiments undertaken here is such that the first indication of tumour growth involves some interpolation from the overall growth curve and represents very early detection. Where detection requires the formation of a readily palpable tumour, an increase in latent period would certainly be observed in our experimental system.

The fact that irradiated tumours frequently regain the exponential rate of growth they displayed before irradiation, and do not commonly display the restraint of growth regularly observed in a pre-irradiated site, still requires elucidation. The results of Experiment (vii) provided no support for the hypothesis adduced in that section, and we are unable to conceive an alternative. The recurrence times of clinical tumours after irradiation with doses giving a high cure rate, are shorter than we should expect for small surviving cell populations growing exponentially with volume doubling times of the order of those commonly encountered in clinical tumours (Suit, Wette and Lindberg, 1967; Porter, E. H., 1967, personal communication). Thus, consideration of a TBE does not appear to be required for the interpretation of clinical tumour response. A similar conclusion has been reached concerning the response of some experimental tumours to single doses of irradiation (Hewitt, 1967).

A general conclusion from the results of experiments on the TBE is that whilst these studies pose interesting problems of interpretation in terms of quantitative mammalian cell radiobiology, they provide no evidence to support an assertion that " indirect " effects of radiation on the stroma of tumours are of importance to their radiotherapeutic eradication.

SUMMARY

1. The volume growth rate of both a rapidly-growing and a slowly-growing murine sarcoma was measurably reduced in the subcutaneous tissue of mouse legs which had been previously exposed to a single dose of 2000 R 250 kv X-rays, compared with the rate in the contralateral unirradiated legs.

2. Growth restraint was similar in legs irradiated 7, 17, 26 and 62 days before the injection of tumour cells. No restraint was observed with an interval of 350 days, but restraint was again apparent when the interval was extended to 450 days. The possibility of a bi-phasic exertion of the effect of pre-irradiation was entertained.

3. The growth restraining effect of pre-irradiation was exerted equally in mice aged 3–4 months and mice aged 12–21 months.

4. Evidence was provided which strongly suggested that pre-irradiation of the site of injection of tumour cells had no influence on the latent phase of subsequent tumour growth. This limitation of the effect to later phases of growth was considered to be associated with an ability of microtumours to grow independently of vascularisation.

5. The subcutaneous injection of two strains of murine leukaemia cells did not give rise to a local tumour in pre-irradiated or normal control sites. The latent period before the development of generalised leukaemia was similar after injection into irradiated and control sites.

6. No restraint of tumour growth was detected in sites pre-irradiated to a dose of 100 or 500 R. The minimum effective dose was between 500 and 1000 R, and there was no measurable discrimination of the effect of dose in the range 1000 to 4000 R.

7. The growth restraining effect of pre-irradiation was fully manifested when the radiation had been delivered to a previous tumour resident in the same site and subsequently excised.

The results are discussed in relation to quantitative cellular radiobiology and the volume response of tumours to irradiation.

We are indebted to Miss Angela Walder for her outstanding management of the breeding and care of the animals used. The expenses of the research were wholly defrayed by the British Empire Cancer Campaign for Research, whose support we gratefully acknowledge.

### REFERENCES

BAKER, D. J., LINDOP, P. J. AND HEWITT, H. B.—(1968) *Br. J. Radiol.*, **41**, 318.

BREUR, K.—(1966) *Eur. J. Cancer*, **2**, 173.

CAMERON, G. R.—(1952) ' Pathology of the Cell '. Edinburgh and London (Oliver and Boyd), p. 440.

CAVANAGH, J. B.—(1968) *Br. J. Radiol.*, **41**, 275.

Frankl, O. and Kimball, G. P.—(1914) *Wien. klin. Wschr.*, **27**, 1448.
Hawkes, M. J., Hill, R. P., Lindop, P. J., Ellis, R. E. and Rotblat, J.—(1968) *Br. J. Radiol.*, **41**, 134.
Hewitt, H. B.—(1963) in ‘Radiation Effects in Physics, Chemistry and Biology’. Proceedings of the 2nd International Congress of Radiation Research, Harrogate, 1962. Edited by Ebert, M. and Howard, A. Amsterdam (North Holland), p. 244.—(1966) *Br. J. Radiol.*, **39**, 19.—(1967) Proceedings of the International Conference on Radiation Biology and Cancer, Kyoto, Japan, 1966. (Radiation Society of Japan), p. 9.
Hewitt, H. B. and Wilson, C. W.—(1961) *Ann. N.Y. Acad. Sci.*, **95**, 818.
Lacassagne, A. and Gricouroff, G.—(1958) ‘Action of Radiation on Tissues’. New York and London (Grune and Stratton), p. 138.
Le Gros Clark, W. E.—(1945) ‘The Tissues of the Body’. 2nd Edition. Oxford (Clarendon Press), p. 190.
Merwin, R., Algire, G. H. and Kaplan, H. S.—(1950) *J. natn. Cancer Inst.*, **11**, 593.
Philp, R. J., Crooks, J., MacGregor, A. G. and McIntosh, J. A. R.—(1966) 3rd International Congress of Radiation Research. Cortina d'Ampezzo, Italy. Abstract 704.
Reinhold, H. S. and De Bree, C.—(1966) 3rd International Congress of Radiation Research. Cortina d'Ampezzo, Italy. Abstract 735.
Stenstrom, K. W., Vermund, H., Mosser, D. G. and Marvin, J. F.—(1955) *Radiat. Res.*, **2**, 180.
Suit, H. D. and Shalek, R. J.—(1963) *J. natn. Cancer Inst.*, **31**, 479.
Suit, H. D., Shalek, R. J. and Wette, R.—(1964) ‘Cellular Radiation Biology’. Baltimore (Williams and Wilkins), p. 514.
Suit, H. D., Wette, R. and Lindberg, R.—(1967) *Radiology*, **88**, 311.
Summers, W. C., Clifton, K. H. and Vermund, H.—(1964) *Radiology*, **82**, 691.
Thomlinson, R. H. and Craddock, E. A.—(1967) *Br. J. Cancer*, **21**, 108.
Urano, M.—(1966) *Nippon Acta radiol.*, **25**, 1326.
Vermund, H.—(1959) *Am. J. Roentg.*, **82**, 678.
Vermund, H., Stenstrom, K. W., Mosser, D. G. and Johnson, E. A.—(1956) *Radiat. Res.*, **5**, 354.
Vermund, H., Stenstrom, K. W., Mosser, D. G. and Loken, M. K.—(1958) *Radiat. Res.*, **8**, 22.
Weinbren, K., Fitschen, W. and Cohen, M.—(1960) *Br. J. Radiol.*, **33**, 419.